# Supplementary material for: Comprehensive Studies on the Regulation of Type 2 Diabetes by Cucurbitane-Type Triterpenoids in Momordica charantia L.: Insights from Network Pharmacology and Molecular Docking and Dynamics
Source: Pharmaceuticals (Basel). 2025 Mar 27;18(4):474. doi: 10.3390/ph18040474 (PMC12030615; doi:10.3390/ph18040474)
Supplement: Supplementary file 1 [file pharmaceuticals-18-00474-s001.zip › pharmaceuticals-3499160-supplementary.pdf]

Supplementary Table

Table S1. The information of the top 10 GO-BP pathways obtained through screening.

NO.	Category	ID	Term	PValue	Genes	FDR
1	GOTERM_BP_DIRECT	GO:0048009	insulin-like growth factor receptor signaling pathway	2.63E-25	PDGFRB, NTRK1, RET, MAP2K1, INSR, EIF2AK3, PIK3R1, EGFR, IGF1R, AR, PIK3CA, KIT, KDR, AKT1, MAPK1, MET, FGFR3, FGFR2, FGFR1, MAPK3	5.65E-22
2	GOTERM_BP_DIRECT	GO:0008286	insulin receptor signaling pathway	1.53E-20	PDGFRB, NTRK1, RET, PTPN1, INSR, PIK3R1, EGFR, IGF1R, PIK3CA, AKT2, KIT, KDR, AKT1, MAPK1, MET, FGFR3, FGFR2, FGFR1, MAPK3	1.64E-17
3	GOTERM_BP_DIRECT	GO:0043410	positive regulation of MAPK cascade	3.88E-19	RET, APP, MAP2K1, ROCK2, INSR, ADRA1B, ADRA2C, ADRA1A, TNF, IGF1R, AR, IL6, KIT, KDR, DRD1, JAK2, DRD2, FGFR3, DRD3, FGFR2, FGFR1	2.78E-16
4	GOTERM_BP_DIRECT	GO:0007173	epidermal growth factor receptor signaling pathway	5.78E-19	PDGFRB, NTRK1, RET, SRC, INSR, BRAF, PTPN11, EGFR, IGF1R, PIK3CA, KIT, KDR, AKT1, MET, FGFR3, FGFR2, FGFR1	3.11E-16
5	GOTERM_BP_DIRECT	GO:0070374	positive regulation of ERK1 and ERK2 cascade	1.29E-18	PDGFRB, NTRK1, APP, MAP2K1, CASR, SRC, HTR2B, HTR2C, BRAF, PTPN11, HTR2A, ADRA1A, TNF, EGFR, ICAM1, TRPV4, KDR, DRD2, FGFR3, FGFR2, MAPK3	5.58E-16
6	GOTERM_BP_DIRECT	GO:0048013	ephrin receptor signaling pathway	3.64E-18	PDGFRB, NTRK1, RET, SRC, MMP2, INSR, PTPN11, MMP9, EGFR, IGF1R, KIT, KDR, MET, FGFR3, FGFR2, FGFR1	1.31E-15
7	GOTERM_BP_DIRECT	GO:0009410	response to xenobiotic stimulus	6.55E-18	NTRK1, RET, HDAC2, CPT1A, MAOB, SRC, MMP2, HTR2B, HTR2A, PTGS2, ACACB, ADRA1A, TNF, HSD11B2, TBXA2R, CASP3, BCL2, PPARG, DRD1, DRD2, TP53, DRD3	2.02E-15
8	GOTERM_BP_DIRECT	GO:0051897	positive regulation of phosphatidylinositol 3-kinase/protein kinase B signal transduction	2.26E-16	PDGFRB, NTRK1, RET, APP, SRC, INSR, F2, TNF, EGFR, MTOR, PIK3CG, IGF1R, PIK3CA, KIT, KDR, JAK2, FGFR3, FGFR1, PPARD	6.08E-14
9	GOTERM_BP_DIRECT	GO:0035791	platelet-derived growth factor receptor-beta signaling pathway	4.79E-16	PDGFRB, NTRK1, RET, PTPN1, INSR, EGFR, IGF1R, KIT, KDR, MET, FGFR3, FGFR2, FGFR1	1.15E-13
10	GOTERM_BP_DIRECT	GO:0007275	multicellular organism development	2.00E-15	NTRK1, PDGFRB, RET, INSR, KIT, KDR, FGFR3, MET, EGFR, FGFR2, FGFR1, IGF1R	4.31E-13

Table S2. The information of the top 10 GO-CC pathways obtained through screening.

NO.	Category	ID	Term	PValue	Genes	FDR
1	GOTERM_CC_DIRECT	GO:0043235	receptor complex	1.12E-18	PDGFRB, NTRK1, RET, APP, SRC, VDR, INSR, ITGB2, PTPRF, EGFR, NR3C2, IGF1R, RXRA, KIT, KDR, PPARG, MET, LDLR, FGFR3, FGFR2, FGFR1	2.60E-16
2	GOTERM_CC_DIRECT	GO:0005886	plasma membrane	7.85E-18	RET, SCARB1, APP, ITGB2, HTR2B, SLC2A1, HTR2C, GRIK1, HTR2A, ADRA1B, ITGAL, ADRA1A, TNF, PTPRF, PIK3CG, IGF1R, ICAM1, HTR7, EDNRB, CA2, AKT2, SCN9A, KDR, AKT1, JAK2, CCR5, PDGFRB, KCNH2, CASR, MAP2K1, MMP2, AVPR2, PRKCD, ADRA2C, F2, GABRG2, AR, PIK3CA, MTNR1B, KCNQ1, KIT, AGTR1, MET, ROCK2, SRC, PIK3R1, SLC5A1, CYP2C19, EGFR, CACNA1H, DPP4, TERT, TBXA2R, MAPK1, DRD1, DRD2, DRD3, KCNJ1, LDLR, DRD4, MAPK3, NTRK1, PTPN1, GABRA1, NOS2, WNT3A, INSR, STAT3, BRAF, ESR1, MTOR, CYP2C9, TRPV4, REN, FGFR3, FGFR2, NFE2L2, FGFR1	9.14E-16
3	GOTERM_CC_DIRECT	GO:0005901	caveola	1.61E-14	SCARB1, SRC, INSR, SLC2A1, HTR2A, PTGS2, ADRA1B, ADRA1A, IGF1R, LIPE, MAPK1, JAK2, MAPK3	1.25E-12
4	GOTERM_CC_DIRECT	GO:0016020	membrane	2.37E-07	RET, SCARB1, APP, ITGB2, SLC2A1, ADRA1B, ITGAL, TNF, PTPRF, PIK3CG, IGF1R, ICAM1, ADAMTS4, LIPE, HTR7, AKT1, JAK2, CCR5, ACP1, PDGFRB, KCNH2, CASR, CPT1A, G6PD, SIGMAR1, AVPR2, GABRG2, AR, MTNR1B, KCNQ1, KIT, AGTR1, MET, TP53, HDAC2, PIK3R1, HMGCR, CYP3A4, EGFR, CACNA1H, DPP4, HSD11B2, NLRP3, DRD3, LDLR, DRD4, PTPN1, GABRA1, HSD3B2, INSR, EIF2AK3, ESR1, MTOR, CYP11B2, TRPV4, BCL2, FGFR3, FGFR2, FGFR1	1.20E-05
5	GOTERM_CC_DIRECT	GO:0009986	cell surface	3.01E-07	KCNH2, NTRK1, SCARB1, APP, CASR, WNT3A, ITGB2, ITGAL, TNF, EGFR, ICAM1, DPP4, TRPV4, CCR5, MET, LDLR, FGFR3, FGFR2	1.20E-05
6	GOTERM_CC_DIRECT	GO:0098978	glutamatergic synapse	3.10E-07	PTPN1, CASR, WNT3A, SRC, SIGMAR1, BRAF, HTR2A, MAPK14, EGFR, CASP3, AKT1, DRD1, JAK2, DRD2, DRD4, FGFR1, MAPK3	1.20E-05
7	GOTERM_CC_DIRECT	GO:0005769	early endosome	1.97E-06	NTRK1, RET, PTPN1, APP, MAP2K1, AKT2, KCNQ1, KDR, MAPK1, SLC5A1, LDLR, MAPK3	6.56E-05
8	GOTERM_CC_DIRECT	GO:0098794	postsynapse	3.55E-06	PTPN1, GABRA1, APP, AKT1, BRAF, JAK2, MET, GABRG2, DRD4, FGFR1	1.03E-04
9	GOTERM_CC_DIRECT	GO:0045121	membrane raft	4.48E-06	DPP4, APP, SRC, KCNQ1, ITGB2, KDR, JAK2, TNF, EGFR, ICAM1	1.16E-04
10	GOTERM_CC_DIRECT	GO:0005737	cytoplasm	7.46E-06	SCARB1, APP, HTR2B, AKR1B1, PYGM, ADRA1B, ADRA1A, PTPRF, PIK3CG, CA1, CA2, CASP3, AKT2, AKT1, JAK2, CCR5, ACP1, PDGFRB, G6PD, PRKCD, ADRA2C, AR, PIK3CA, KCNQ1, PPARG, TP53, HDAC2, ROCK2, SRC, NR1I3, PIK3R1, CYP2C19, CYP3A4, PTGS2, EGFR, CYP27B1, TERT, MAPK1, NLRP3, RXRG, MAPK3, PTPN1, HSD3B2, CHKA, NOS2, VDR, IDH1, STAT3, EIF2AK3, PTPN11, BRAF, MAPK14, ESR1, MTOR, CYP2C9, BCL2, FGFR2, NFE2L2	1.74E-04

Table S3. The information of the top 10 GO-MF pathways obtained through screening.

NO.	Category	ID	Term	PValue	Genes	FDR
1	GOTERM_MF_DIRECT	GO:0004713	protein tyrosine kinase activity	1.12E-20	PDGFRB, NTRK1, RET, MAP2K1, CHKA, SRC, INSR, EIF2AK3, BRAF, EGFR, MTOR, IGF1R, KIT, KDR, JAK2, MET, FGFR3, FGFR2, FGFR1	5.25E-18
2	GOTERM_MF_DIRECT	GO:0035401	histone H3Y41 kinase activity	5.30E-19	PDGFRB, NTRK1, RET, MAP2K1, CHKA, SRC, INSR, PRKCD, EIF2AK3, EGFR, IGF1R, KIT, KDR, JAK2, MET, FGFR3, FGFR2, FGFR1	8.26E-17
3	GOTERM_MF_DIRECT	GO:0140801	histone H2AXY142 kinase activity	5.30E-19	PDGFRB, NTRK1, RET, MAP2K1, CHKA, SRC, INSR, PRKCD, EIF2AK3, EGFR, IGF1R, KIT, KDR, JAK2, MET, FGFR3, FGFR2, FGFR1	8.26E-17
4	GOTERM_MF_DIRECT	GO:0042802	identical protein binding	5.37E-18	APP, MAOB, SLC2A1, HTR2C, HTR2A, TNF, ACACB, EGFR, PIK3CG, IGF1R, DPP4, RXRA, TERT, KDR, AKT1, MAPK1, NLRP3, JAK2, CCR5, DRD2, LDLR, DRD4, MAPK3, KCNH2, NTRK1, CASR, CPT1A, G6PD, WNT3A, IDH1, INSR, SIGMAR1, STAT3, EIF2AK3, BRAF, MMP9, ESR1, MTOR, IL6, TRPV4, BCL2, PPARG, MET, FGFR3, TP53, FGFR2, FGFR1	6.27E-16
5	GOTERM_MF_DIRECT	GO:0004879	nuclear receptor activity	5.44E-16	VDR, NR1I3, STAT3, PIK3R1, ESR1, NR3C2, RXRB, AR, RXRA, PPARG, PPARA, RXRG, PPARD	5.08E-14
6	GOTERM_MF_DIRECT	GO:0036332	placental growth factor receptor activity	8.41E-15	NTRK1, PDGFRB, RET, INSR, KIT, KDR, FGFR3, MET, EGFR, FGFR2, FGFR1, IGF1R	2.80E-13
7	GOTERM_MF_DIRECT	GO:0005018	platelet-derived growth factor alpha-receptor activity	8.41E-15	NTRK1, PDGFRB, RET, INSR, KIT, KDR, FGFR3, MET, EGFR, FGFR2, FGFR1, IGF1R	2.80E-13
8	GOTERM_MF_DIRECT	GO:0008288	boss receptor activity	8.41E-15	NTRK1, PDGFRB, RET, INSR, KIT, KDR, FGFR3, MET, EGFR, FGFR2, FGFR1, IGF1R	2.80E-13
9	GOTERM_MF_DIRECT	GO:0060175	brain-derived neurotrophic factor receptor activity	8.41E-15	NTRK1, PDGFRB, RET, INSR, KIT, KDR, FGFR3, MET, EGFR, FGFR2, FGFR1, IGF1R	2.80E-13
10	GOTERM_MF_DIRECT	GO:0005020	stem cell factor receptor activity	8.41E-15	NTRK1, PDGFRB, RET, INSR, KIT, KDR, FGFR3, MET, EGFR, FGFR2, FGFR1, IGF1R	2.80E-13

Table S4. The information of the top 30 KEGG pathways obtained through screening.

NO.	Category	ID	Term	PValue	Genes	FDR
1	KEGG_PATHWAY	hsa05200	Pathways in cancer	2.55E-25	RET, HDAC2, ROCK2, SLC2A1, PIK3R1, PTGS2, EGFR, IGF1R, RXRB, RXRA, EDNRB, TERT, CASP3, AKT2, AKT1, MAPK1, JAK2, RXRG, MAPK3, PDGFRB, NTRK1, MAP2K1, NOS2, MMP1, WNT3A, MMP2, STAT3, BRAF, F2, MMP9, ESR1, MTOR, AR, IL6, PIK3CA, KIT, BCL2, AGTR1, PPARG, MET, FGFR3, TP53, FGFR2, FGFR1, PPARD, NFE2L2	3.26E-23
2	KEGG_PATHWAY	hsa05230	Central carbon metabolism in cancer	1.97E-23	PDGFRB, NTRK1, RET, MAP2K1, G6PD, IDH1, SLC2A1, PIK3R1, EGFR, MTOR, GCK, PIK3CA, AKT2, KIT, AKT1, MAPK1, MET, FGFR3, TP53, FGFR2, FGFR1, MAPK3	1.26E-21
3	KEGG_PATHWAY	hsa01521	EGFR tyrosine kinase inhibitor resistance	1.02E-20	PDGFRB, MAP2K1, SRC, STAT3, BRAF, PIK3R1, EGFR, MTOR, IGF1R, IL6, PIK3CA, AKT2, KDR, BCL2, AKT1, MAPK1, JAK2, MET, FGFR3, FGFR2, MAPK3	4.36E-19
4	KEGG_PATHWAY	hsa05417	Lipid and atherosclerosis	1.67E-20	ROCK2, SRC, PIK3R1, TNF, ICAM1, RXRB, RXRA, CASP3, AKT2, AKT1, MAPK1, NLRP3, JAK2, LDLR, RXRG, MAPK3, MMP1, STAT3, EIF2AK3, MAPK14, MMP9, CYP2C9, IL6, PIK3CA, IL1B, BCL2, PPARG, TP53, NFE2L2	5.35E-19
5	KEGG_PATHWAY	hsa05205	Proteoglycans in cancer	9.63E-18	ROCK2, SRC, PIK3R1, TNF, EGFR, IGF1R, CASP3, AKT2, KDR, AKT1, MAPK1, MAPK3, MAP2K1, WNT3A, MMP2, STAT3, PTPN11, BRAF, MAPK14, MMP9, ESR1, MTOR, PIK3CA, MET, TP53, FGFR1	2.47E-16
6	KEGG_PATHWAY	hsa05215	Prostate cancer	7.36E-15	PDGFRB, MAP2K1, BRAF, PIK3R1, MMP9, EGFR, MTOR, IGF1R, AR, PIK3CA, AKT2, BCL2, AKT1, MAPK1, TP53, FGFR2, FGFR1, MAPK3	1.57E-13
7	KEGG_PATHWAY	hsa01522	Endocrine resistance	8.83E-15	MAP2K1, SRC, MMP2, BRAF, PIK3R1, MAPK14, ESR1, MMP9, EGFR, MTOR, IGF1R, PIK3CA, AKT2, BCL2, AKT1, MAPK1, TP53, MAPK3	1.61E-13
8	KEGG_PATHWAY	hsa04933	AGE-RAGE signaling pathway in diabetic complications	1.25E-14	MMP2, PRKCD, STAT3, PIK3R1, MAPK14, TNF, ICAM1, IL6, PIK3CA, IL1B, CASP3, AKT2, BCL2, AGTR1, AKT1, MAPK1, JAK2, MAPK3	2.01E-13
9	KEGG_PATHWAY	hsa05223	Non-small cell lung cancer	2.25E-14	RET, MAP2K1, STAT3, BRAF, PIK3R1, EGFR, RXRB, RXRA, PIK3CA, AKT2, AKT1, MAPK1, MET, RXRG, TP53, MAPK3	3.19E-13
10	KEGG_PATHWAY	hsa04015	Rap1 signaling pathway	4.05E-14	PDGFRB, MAP2K1, SRC, INSR, ITGB2, BRAF, PIK3R1, ITGAL, MAPK14, EGFR, IGF1R, PIK3CA, AKT2, KIT, KDR, AKT1, MAPK1, DRD2, MET, FGFR3, FGFR2, FGFR1, MAPK3	5.18E-13
11	KEGG_PATHWAY	hsa04931	Insulin resistance	4.69E-14	PTPN1, CPT1A, INSR, PRKCD, STAT3, SLC2A1, PYGM, PTPN11, PIK3R1, ACACB, TNF, PTPRF, MTOR, IL6, PIK3CA, AKT2, AKT1, PPARA	5.46E-13
12	KEGG_PATHWAY	hsa05207	Chemical carcinogenesis - receptor activation	5.46E-13	MAP2K1, SRC, VDR, NR1I3, STAT3, PIK3R1, CYP3A4, ESR1, EGFR, MTOR, RXRB, AR, RXRA, PIK3CA, AKT2, BCL2, AKT1, MAPK1, JAK2, PPARA, RXRG, MAPK3	5.82E-12
13	KEGG_PATHWAY	hsa05226	Gastric cancer	8.54E-13	MAP2K1, WNT3A, BRAF, PIK3R1, EGFR, MTOR, RXRB, RXRA, TERT, PIK3CA, AKT2, BCL2, AKT1, MAPK1, MET, RXRG, TP53, FGFR2, MAPK3	8.41E-12
14	KEGG_PATHWAY	hsa04920	Adipocytokine signaling pathway	5.28E-12	CPT1A, STAT3, SLC2A1, PTPN11, ACACB, TNF, MTOR, RXRB, RXRA, AKT2, AKT1, JAK2, PPARA, RXRG	4.83E-11
15	KEGG_PATHWAY	hsa04010	MAPK signaling pathway	6.27E-12	PDGFRB, NTRK1, RET, MAP2K1, INSR, BRAF, MAPK14, TNF, EGFR, CACNA1H, IGF1R, IL1B, CASP3, AKT2, KIT, KDR, AKT1, MAPK1, MET, FGFR3, TP53, FGFR2, FGFR1, MAPK3	5.35E-11
16	KEGG_PATHWAY	hsa04151	PI3K-Akt signaling pathway	6.72E-12	RET, PIK3R1, EGFR, PIK3CG, IGF1R, RXRA, AKT2, KDR, AKT1, MAPK1, JAK2, MAPK3, PDGFRB, NTRK1, MAP2K1, INSR, MTOR, IL6, PIK3CA, KIT, BCL2, MET, FGFR3, TP53, FGFR2, FGFR1	5.37E-11
17	KEGG_PATHWAY	hsa05218	Melanoma	9.28E-12	PDGFRB, MAP2K1, BRAF, PIK3R1, EGFR, IGF1R, PIK3CA, AKT2, AKT1, MAPK1, MET, TP53, FGFR1, MAPK3	6.99E-11

18	KEGG_PATHWAY	hsa04066	HIF-1 signaling pathway	1.05E-11	MAP2K1, NOS2, INSR, STAT3, SLC2A1, PIK3R1, EGFR, MTOR, IGF1R, IL6, PIK3CA, AKT2, BCL2, AKT1, MAPK1, MAPK3	7.49E-11
19	KEGG_PATHWAY	hsa05160	Hepatitis C	2.56E-11	SCARB1, MAP2K1, STAT3, EIF2AK3, BRAF, PIK3R1, TNF, EGFR, RXRA, PIK3CA, CASP3, AKT2, AKT1, MAPK1, PPARA, LDLR, TP53, MAPK3	1.72E-10
20	KEGG_PATHWAY	hsa05216	Thyroid cancer	3.06E-11	RXRB, NTRK1, RET, MAP2K1, RXRA, MAPK1, BRAF, PPARG, RXRG, TP53, MAPK3	1.96E-10
21	KEGG_PATHWAY	hsa05161	Hepatitis B	3.82E-11	MAP2K1, SRC, STAT3, BRAF, PIK3R1, MAPK14, TNF, MMP9, IL6, PIK3CA, CASP3, AKT2, BCL2, AKT1, MAPK1, JAK2, TP53, MAPK3	2.33E-10
22	KEGG_PATHWAY	hsa04919	Thyroid hormone signaling pathway	5.53E-11	MAP2K1, HDAC2, SRC, SLC2A1, PIK3R1, ESR1, MTOR, RXRB, RXRA, PIK3CA, AKT2, AKT1, MAPK1, RXRG, TP53, MAPK3	3.22E-10
23	KEGG_PATHWAY	hsa05167	Kaposi sarcoma-associated herpesvirus infection	8.26E-11	MAP2K1, SRC, STAT3, PIK3R1, PTGS2, MAPK14, MTOR, PIK3CG, ICAM1, IL6, PIK3CA, CASP3, AKT2, AKT1, MAPK1, JAK2, CCR5, TP53, MAPK3	4.44E-10
24	KEGG_PATHWAY	hsa04625	C-type lectin receptor signaling pathway	8.33E-11	SRC, PRKCD, PTPN11, PIK3R1, PTGS2, MAPK14, TNF, IL6, PIK3CA, IL1B, AKT2, AKT1, NLRP3, MAPK1, MAPK3	4.44E-10
25	KEGG_PATHWAY	hsa05219	Bladder cancer	9.41E-11	MAP2K1, MMP1, SRC, MMP2, MAPK1, BRAF, FGFR3, TP53, MMP9, EGFR, MAPK3	4.76E-10
26	KEGG_PATHWAY	hsa04072	Phospholipase D signaling pathway	9.68E-11	PDGFRB, MAP2K1, INSR, AVPR2, PTPN11, PIK3R1, F2, EGFR, MTOR, PIK3CG, PIK3CA, AKT2, KIT, AGTR1, AKT1, MAPK1, MAPK3	4.76E-10
27	KEGG_PATHWAY	hsa05163	Human cytomegalovirus infection	1.09E-10	MAP2K1, ROCK2, SRC, STAT3, PIK3R1, PTGS2, MAPK14, TNF, EGFR, MTOR, IL6, PIK3CA, IL1B, CASP3, AKT2, AKT1, MAPK1, CCR5, TP53, MAPK3	5.02E-10
28	KEGG_PATHWAY	hsa04020	Calcium signaling pathway	1.10E-10	PDGFRB, NTRK1, RET, NOS2, HTR2B, HTR2C, HTR2A, ADRA1B, ADRA1A, EGFR, CACNA1H, HTR7, EDNRB, TBXA2R, KDR, AGTR1, DRD1, MET, FGFR3, FGFR2, FGFR1	5.02E-10
29	KEGG_PATHWAY	hsa04917	Prolactin signaling pathway	1.16E-10	MAP2K1, SRC, STAT3, PIK3R1, MAPK14, ESR1, GCK, PIK3CA, AKT2, AKT1, MAPK1, JAK2, MAPK3	5.12E-10
30	KEGG_PATHWAY	hsa05214	Glioma	2.66E-10	PDGFRB, MAP2K1, BRAF, PIK3R1, EGFR, MTOR, IGF1R, PIK3CA, AKT2, AKT1, MAPK1, TP53, MAPK3	1.10E-09
